# Comparative design of artificial ochratoxin A antigens: conjugation strategies and immunogenicity evaluation for antibody production

**DOI:** 10.3389/fvets.2026.1734152

**Published:** 2026-04-30

**Authors:** Li Han, Yuetao Li, Haocheng Zhang, Yidan Wang, Jinqing Jiang, Renfeng Li, Guoying Fan, Yanhong Cui, Yongqiang Wang, Ziliang Wang, Changzhong Liu, Wen Chen

**Affiliations:** 1 College of Animal Science and Veterinary Medicine, Henan Institute of Science and Technology, Xinxiang, China; 2 Sumy International School, Henan Institute of Science and Technology, Xinxiang, China; 3 College of Animal Science and Technology, Henan Agricultural University, Zhengzhou, China

**Keywords:** antibody production, artificial antigen, comparative design, conjugation strategies, immune evaluation, immunogenicity, ochratoxin A

## Abstract

Ochratoxin A (OTA) is a common fungal toxin contaminant in feed that is primarily produced by fungi belonging to the *Aspergillus* and *Penicillium* genera. In animal husbandry, the long-term ingestion of OTA-contaminated feed by animals can lead to severe consequences, such as reduced production performance, immunosuppression, and kidney and liver damage. Therefore, the development of efficient OTA detection technology is crucial for ensuring animal health and enhancing farming efficiency. This study aimed to design and modify the molecular structure of OTA, compare the preparation and identification of two artificial antigens, and select the optimal artificial antigen. Based on the molecular structure of OTA, the OTA hapten was activated via the N-hydroxysuccinimide active ester (NHS) method to generate an intermediate product known as OTA-carboxylic acid ester. This intermediate was subsequently conjugated with bovine serum albumin (BSA) via the coupling agents N, N′-dicyclohexylcarbodiimide (DCC) or 1-(3-dimethylaminopropyl)-3-ethylcarbodiimide hydrochloride (EDC) to prepare new and effective artificial antigens. Additionally, ovalbumin (OVA) was used to prepare detection antigens. The structures of the prepared artificial antigens were physically and chemically characterized via instrumental analysis techniques such as ultraviolet (UV) and infrared (IR) spectroscopy. Mice were immunized with artificial antigens to explore their immunogenicity. The results revealed successful conjugation of both artificial antigens with molecular binding ratios of OTA to BSA of 10.88:1 and 7.84:1. The immunized mice exhibited corresponding responses, and the titers of the mouse polyclonal antisera (pAbs) reached 1:3.2 × 10^3^, the OTA pAb that was prepared from two artificial antigens was able to recognize OTA with 100% accuracy. Among them, the mice immunized with the artificial antigen OTA-BSA (EDC) exhibited the best sensitivity, with a half-maximal inhibitory concentration (IC_50_) of 11.40 ng/mL. Although the cross-reactivity rates with ochratoxin B (OTB) and ochratoxin C (OTC) were relatively high at 86.69% and 62.02%, respectively, the cross-reactivity rates with other compounds were less than 0.3%. In conclusion, this study successfully prepared two artificial antigens and selected the OTA-BSA (EDC) group as the optimal artificial antigen via identification. These results provide the antigenic foundation for the preparation of OTA monoclonal antibodies (mAbs) and the establishment of immunological analysis methods.

## 1 Introduction

Ochratoxin A (OTA) is a common secondary metabolite produced by several *Aspergillus* and *Penicillium* species. It appears in various agricultural products during agricultural production and storage, whereby it contaminates a wide range of feeds and foods (1, 2). Grains such as corn, millet, wheat, barley, and oat are the primary crops contaminated by OTA (3–6), whereas grapes, olives, figs, coffee beans, nuts, and spices are also susceptible to contamination (7, 8). Additionally, OTA is found in animal products such as meat and dairy, as the toxin can be transferred and accumulate when animals consume contaminated feed (9, 10). It is estimated that approximately 30% of global feed is contaminated with OTA to varying degrees, thereby causing billions of dollars in economic losses to the animal husbandry industry each year (11–13). OTA, which is a derivative of phenylalanine combined with isocoumarin, is distinguished by the presence of a chlorine substituent in its structure, which likely contributes to its potent toxicity (14). The molecular formula of OTA is C_20_H_18_ClNO_6_, it is a white, odorless, thermally stable crystalline solid that is soluble in organic solvents such as chloroform and methanol but only slightly soluble in water. Due to its high stability under temperature variations and hydrolytic conditions, it is difficult to completely remove OTA from contaminated feed and food using conventional processing methods (2, 15). OTA has garnered global attention both for the significant economic losses that it causes and for the major risks that it poses to human and animal health. According to the World Health Organization’s International Agency for Research on Cancer (IARC), OTA was classified as a Group 2B carcinogen (possibly carcinogenic to humans) on October 27, 2017 (16). Toxicological studies have revealed that OTA exhibits strong toxicity, including nephrotoxicity, hepatotoxicity, teratogenicity, carcinogenicity, mutagenicity, neurotoxicity, and immunotoxicity (17–20). Currently, the European Commission has established maximum limits for OTA content in feeds and foods to ensure safety, such as 5 μg/kg for raw cereals and roasted coffee; 3.0 μg/kg for cereals and cereal products for human consumption; 2 μg/kg for wine and grape juice; and 0.5 μg/kg for infant foods and cereal-based foods for young children (21). Therefore, there is an urgent need to develop analytical methods with high sensitivity and specificity for detecting OTA in feeds and foods.

There are numerous techniques available for the detection of mycotoxins, and the typical analytical methods that are used to detect OTA in feed and food samples primarily include HPLC with a fluorescence detector (FLD) (22–24) or liquid chromatography/tandem mass spectrometry (LC–MS/MS) (25–27). Although chromatographic methods are sensitive and accurate, they are also very expensive to perform, with high detection costs, low throughput, and high time consumption; moreover, they require skilled operators, complex sample pretreatment, and costly instrumentation, thus making them unsuitable for routine screening in feed and food safety monitoring. In recent years, the application of immunological detection technology has become increasingly widespread for the residual analysis of small-molecule pollutants such as biotoxins, pesticides, and antibiotics (28). Currently, immunoassay methods have become the mainstream technology for OTA detection in feeds because of their simple operation, low costs, and suitability for large-scale screening. Immunoassays are typically quick to perform, with high sensitivity and specificity, simple sample preparation, and high throughput characteristics, thereby resulting in low detection costs per sample (29–31). Many studies have reported the use of immunoassay methods with different signal readouts for OTA detection, such as chemiluminescence (32), time-resolved fluorescence (33), and electrochemistry (34). Additionally, numerous indirect competitive ELISAs (ic-ELISAs) and direct competitive ELISAs (dc-ELISAs) based on polyclonal antibodies (pAbs) or monoclonal antibodies (mAbs) have been developed for the detection of OTA in feed and food samples.

Immunological detection techniques typically rely on monoclonal antibodies (mAbs) as core reagents. The quality of highly specific mAbs directly determines the sensitivity and accuracy of the detection techniques, and the foundation for high-quality mAb core reagents is based on the preparation of high-quality artificial antigens (35). Traditional methods for preparing artificial antigens experience issues such as unstable coupling efficiency and significant differences in immunogenicity, which severely limit the acquisition of high-quality antibodies. Therefore, the synthesis of OTA artificial antigens has become particularly important (36). In summary, as a traditional method, immunodetection does not require special equipment, is suitable for onsite use, and is applicable for high-throughput screening. Moreover, it is considered to be a suitable detection tool, and its development and application have rapidly progressed in recent years (37). This study focused on the molecular structure design of OTA, the preparation of artificial antigens, and immunogenicity research. The aim of this study was to develop and prepare anti-OTA mAbs with high affinity, sensitivity, and specificity, thus providing a solid foundation for the establishment of immunological detection methods such as test kits and test strips. This research not only provides new technical methods for feed safety monitoring but also offers important references for the preparation of artificial antigens for other mycotoxins.

## 2 Materials and methods

### 2.1 Reagents and materials

The standards for OTA, OTB, and OTC were purchased from Beijing Kezhan Biotechnology Co., Ltd. (Beijing, China). Deoxynivalenol (DON), zearalenone (ZEN), and aflatoxin B1 (AFB1) were used to test cross-reactivity, and these Sigma products were purchased from the Yingke Reagent Consumables Business Department in Zhengzhou (Zhengzhou, Henan). N-hydroxysuccinimide (NHS), N, N-dicyclohexylcarbodiimide (DCC), anhydrous tetrahydrofuran (THF), N, N-dimethylformamide (DMF), and 1-ethyl-3-(3-dimethylaminopropyl) carbodiimide hydrochloride (EDC) were Pierce products that were purchased from Anyang Baileji Biotechnology Co., Ltd. (Anyang, Henan). Tetramethylbenzidine (TMB) was purchased from Shanghai Wulian Chemical Plant (Shanghai, China). Chicken ovalbumin (OVA, MW: 45000), bovine serum albumin (BSA, MW: 66000), horseradish peroxidase (HRP), Freund’s incomplete adjuvant (FIA), and Freund’s complete adjuvant (FCA) were Gibco products that were purchased from Anyang Taike Biotechnology Co., Ltd. (Anyang, Henan). Sheep anti-mouse IgG antibody, which is also known as enzyme-linked secondary antibody (GaMIgG HRP), was purchased from Henan Jingge Trading Co., Ltd. (Xinxiang, Henan). Potassium bromide (KBr) was purchased from Tianjin Guangfu Fine Chemical Plant (Tianjin, China).

MD25 dialysis bags (8,000 ~ 14,000 D) and 96-well microplates were purchased from Shanghai Huamei Biotechnology Company (Shanghai, China). Phosphate buffer solution (PBS), carbonate buffer solution (CBS), washing solution (PBST), blocking solution (SPBST), substrate chromogenic solution (TMB), and termination solution (2 M H_2_SO_4_) were synthesized in our laboratory. Female BALB/c mice (6 ~ 8-weeks-old) were purchased from the Experimental Animal Center of Zhengzhou University School of Medicine and raised under strictly controlled conditions in our laboratory animal room (animal license number: SCXK (Yu) 2010–0002).

### 2.2 Instruments and equipment

The Varioskan Flash multifunctional microplate reader and Sorvall Legend Micro 17 microcentrifuge were purchased from Thermo Fisher Scientific (Waltham, MA, USA). A Tensor 27 infrared spectrometer was purchased from Bruker (Wiesbaden, Germany). The DU 800 UV/visible spectrophotometer was purchased from Beckman Coulter (Gottingen, Germany). The 769YP-15A powder tablet press was purchased from Tianjin Keqi High Tech Company (Tianjin, China). The CJ-78-1 digital display constant-temperature magnetic stirrer was purchased from Shanghai Longyue Instrument Equipment Co., Ltd. (Shanghai, China). The BS124S electronic analytical balance was purchased from Beijing Sartorius Instrument Systems Co., Ltd. (Beijing, China). The DHP-9082 electric thermostatic incubator was purchased from Shanghai Yiheng Scientific Instrument Co., Ltd. (Shanghai, China). The immunogen emulsifying machine was purchased from Foshan Yusen Medical Equipment Co., Ltd. (Foshan, MA, USA). The ultrapure water instrument was purchased from Shanghai Danding International Trading Co., Ltd. (Shanghai, China). The MTN-2800D nitrogen-blowing concentration device was purchased from Millipore Corporation (Boston, MA, USA).

### 2.3 Test methods

#### 2.3.1 Preparation of the OTA artificial antigen

**Figure 1:**
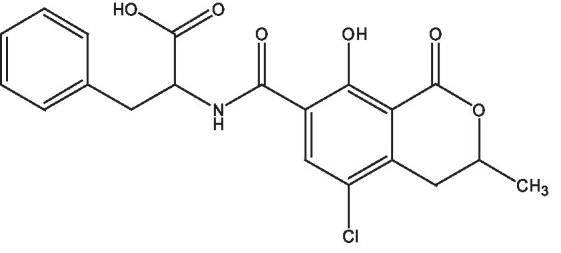
Molecular structure of OTA.

Based on the unique active sites and groups in the molecular structure of OTA (Figure 1), OTA was selected as the starting material for molecular structure modification and artificial antigen preparation. The active ester method was primarily adopted to react the carboxyl group (–COOH) of OTA with the hydroxyl group (-OH) on the NHS molecule, thus introducing the active group carboxylate (–COON). Afterwards, under the action of the coupling agents DCC or EDC, the introduced –COON was coupled with the amino group (–NH_2_) on the carrier protein (BSA) in the form of a single amide bond (–CONH–), thus preparing the artificial antigen (OTA-BSA). The detection antigen (OTA-OVA) can be prepared via the same method.

**Figure 2:**
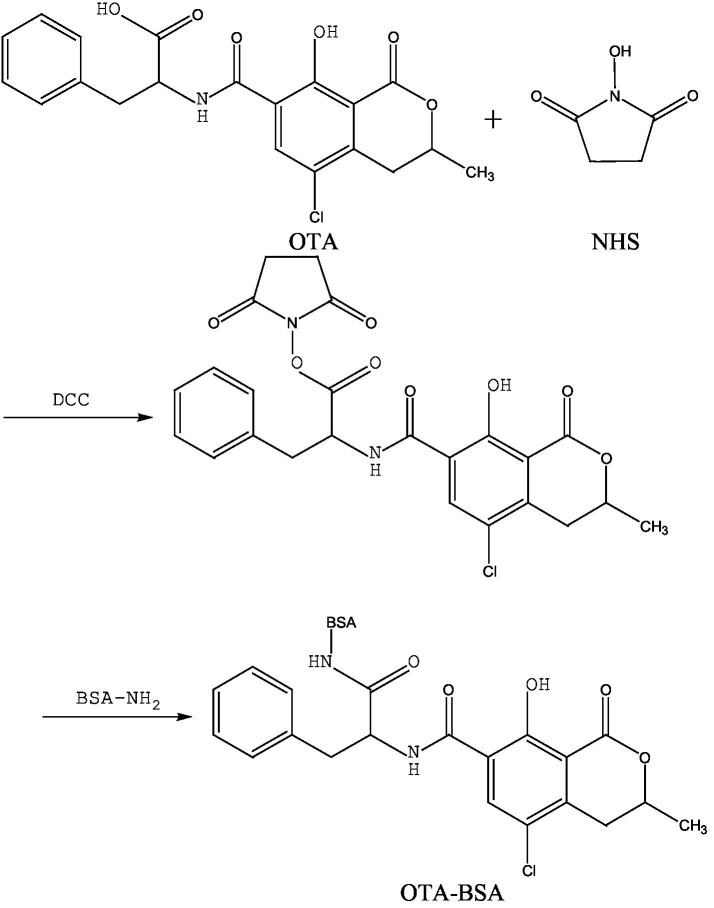
The synthetic route of OTA-BSA by NHS-DCC method.

The N-hydroxy succinimide-N, N-dicyclohexylcarbodiimide (NHS-DCC) method. In this experiment, under the action of the coupling agent DCC, the introduced -COON- group was coupled with the amino group (-NH_2_) on the carrier protein (BSA) in the form of -CONH-, thereby preparing the artificial antigen (OTA-BSA). The specific operational steps were based on the method described by Venkataramana, et al. (38), Guo et al. (39), Yexuan Ren et al. (40), with some modifications. Briefly, 3.63 mg of OTA, 2.1 mg of NHS, and 7.5 mg of DCC were weighed according to the molar ratio (molar ratio OTA: NHS: DCC = 1:2:4) and dissolved in 0.5 mL of DMF. The mixture was stirred and reacted in the dark at room temperature for approximately 10 h (50% of the DCC was first added, and the remaining 50% was added after approximately 6 h). This liquid was defined as the OTA hapten. Ten milligrams of BSA was dissolved in 1 mL of 0.13 mol/L NaHCO_3_ solution to prepare a BSA activation solution. Under ice-bath stirring conditions, the OTA hapten was added dropwise to the BSA solution, with this process occurring for approximately 10 min. Afterwards, the mixture was stirred and reacted in the dark at room temperature for a coupling time of 4 h. The reaction product was dialyzed in a 0.01 mol/L PBS solution for 72 h to remove free OTA and other small molecules, thus resulting in the artificial antigen (OTA-BSA), which was stored at −20 °C for future use. The detection antigen (OTA-OVA) was prepared via the same method. The synthesis route is shown in Figure 2.

**Figure 3:**
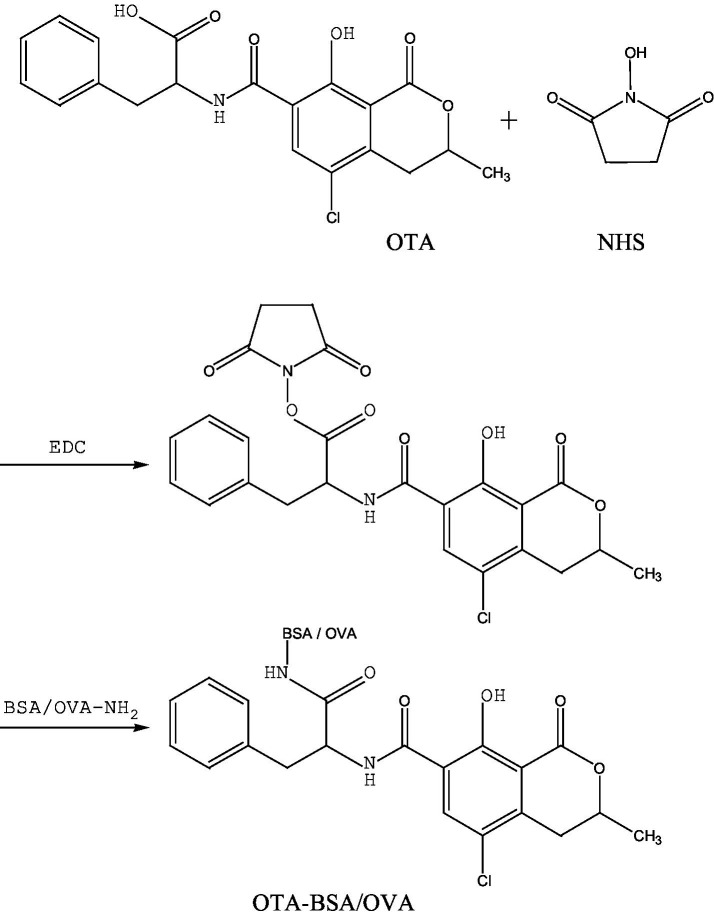
The synthetic route of OTA-BSA/OVA by NHS-EDC method.

The N-hydroxy succinimide-1-ethyl-3-(3-dimethylaminopropyl) carbodiimide hydrochloride (NHS-EDC) method. In this experiment, under the action of the coupling agent EDC, the introduction of -COON was coupled with -NH_2_ on the carrier protein (BSA) in the form of -CONH- to prepare the artificial antigen (OTA-BSA). The specific operational steps were based on and improved upon the method described by Venkataramana, et al. (38), Guo et al. (39), Yexuan Ren et al. (40). Briefly, 3.63 mg of OTA, 2.1 mg of NHS, and 7.5 mg of EDC were weighed according to the molar ratio (molar ratio OTA: NHS: EDC = 1:2:4) and dissolved in 1 mL of THF. The reaction was performed at room temperature for 6 h and then blown dry with nitrogen for approximately 30 min. The product was subsequently dissolved in 1 mL of DMF and reacted for 2 or 4 h. This mixture was defined as the OTA hapten. Afterwards, 10 mg of BSA was dissolved in 1 mL of 0.13 mol/L NaHCO_3_ solution to prepare the BSA activation solution. Under ice-bath stirring conditions, the OTA hapten was added dropwise to the BSA solution for approximately 10 min. The coupling time was 4 h, followed by stirring and reacting at room temperature overnight in the dark. Dialysis was performed as in the previous experiment, after which the artificial antigen (OTA-BSA) was stored at −20 °C for future use. The same method was used to prepare the detection antigen (OTA-OVA). The synthesis route is shown in Figure 3.

#### 2.3.2 Identification of the physical and chemical properties of artificial antigens

##### 2.3.2.1 UV scanning identification

The protein concentrations of the two artificial antigens were diluted with PBS to a concentration of 1 mg/mL. BSA was prepared in a solution with a concentration of 1 mg/mL. To facilitate the scanning of the UV absorption peak of the OTA standard substance, a concentration of 1 mg/mL was prepared with anhydrous methanol, and the blank control was prepared with PBS. Using a DU 800 UV/visible spectrophotometer, the absorption peaks of five substances, including PBS, BSA, OTA, OTA-BSA (DCC), and OTA-BSA (EDC), were measured via scanning in the wavelength range of 200 ~ 450 nm. The successful coupling of these two artificial antigens was analyzed to determine the coupling status of the conjugates (41).

For the determination of the molecular binding ratio of the artificial antigens in this experiment, UV absorption spectroscopy was used to measure the absorbance values of PBS, BSA, OTA, OTA-BSA (DCC), and OTA-BSA (EDC) at 332 nm and 276 nm. The concentrations of these four solutions were all 1 mg/mL. The binding ratio (O/P) between OTA and the carrier protein (BSA) was subsequently calculated according to the following formula (42):


OP=AOTA−BSA332×KBSA276−AOTA−BSA276×KBSA332AOTA−BSA276×KOTA332−AOTA−BSA332×KOTA276 (1)

K=A×M/C (2)

Where *K* is the molar extinction coefficient of a substance at its maximum absorption peak, *C* is the concentration, *M* is the molar mass, and *A* is the absorbance value.

##### 2.3.2.2 IR identification

A freeze dryer was used to freeze dry two artificial antigens for 48 h, after which 1 mg of each antigen was weighed and added to 200 ~ 250 mg of dried KBr. The samples were ground and mixed in an agate mortar under infrared light irradiation. The samples were thoroughly placed into the mold, after which the mold was placed in the center of the tablet press (with a pressure not exceeding 15 MPa and a tablet pressing time not exceeding 2 min) to obtain 1 mm thick KBr transparent sample tablets (43). The same method was used to separately prepare KBr samples of OTA and BSA.

#### 2.3.3 Identification of the immunological characteristics of artificial antigens

##### 2.3.3.1 Preparation of OTA pAbs

Six-week-old healthy female BALB/c mice were immunized with two types of artificial antigens, with one group being immunized with each artificial antigen (consisting of five mice in each group) for a total of two groups. When a low-dose, long interval immunization regimen was adopted, 1 mL of artificial antigen was initially prepared with sterile PBS. The immunization dose was 30 μg/animal, with 5 immunizations and a 1-month interval being utilized between each immunization. The antigen was subcutaneously injected into the back at 4 points. With the exception of the first immunization with a mixed emulsification of FCA, the other immunizations involved FIA. Twenty-one days after the fifth (last) immunization, the tail tip was cut with small scissors for blood collection, and the supernatant was collected via centrifugation to obtain OTA pAbs. During blood collection, the collected serum was diluted 1:100 with diluted PBS and centrifuged for 10 min at 3000 r/min. After centrifugation, the supernatant was stored in a refrigerator at 4 °C for later use.

##### 2.3.3.2 Determination of the titer of OTA pAbs

The indirect ELISA method was used to determine the titer of OTA pAbs. Using an A450 nm enzyme-linked immunosorbent assay (ELISA) reader, the effective positive potency was evaluated by determining that the A450 nm value of the test well was ≥2.1 times the A450 nm value of the negative well. The operating procedure followed the conventional operating method (44).

##### 2.3.3.3 Sensitivity determination of OTA pAbs

Sensitivity determination of the OTA pAbs was performed via the ic-ELISA method. The working concentration of OTA pAbs during determination was measured via the concentration at an A450 nm ≈ 1.0 during potency determination. The experimental results were used to calculate the inhibition rate of the anti-OTA pAb; additionally, an inhibition curve was plotted and fitted, after which the correlation coefficient and regression equation were obtained from the inhibition curve. The IC_50_ of OTA was calculated and used as the result of the sensitivity determination. The operating procedure followed conventional operating methods (45, 46).

##### 2.3.3.4 Specific identification of the OTA pAb

A cross-reaction assay was used to identify the specificity of the OTA pAb. The IC_50_ values of the pAb against functional analogs, including OTB, OTC, DON, ZEN, and AFB1, were determined via the ic-ELISA method. In addition, the cross-reactivity rate (CR %) was calculated as the percentage of the IC_50_ of the pAb on OTA divided by the IC_50_ of the pAb on other functional analogs. The utilized formula for this calculation is as follows (47):


$$\mathrm{CR}\%[47]=IC_{50}=\frac{\begin{array}{c} IC_{50}\;\text{VALUE}\;\\ \text{OF}\;\mathrm{PAB}\;\text{VERSUS}\;\\ \mathrm{OTA} \end{array}}{\begin{array}{c} IC_{50}\;\text{VALUE OF}\;\\ \mathrm{PAB}\;\text{VERSUS OTHER}\;\\ \text{FUNCTIONAL ANALOGS} \end{array}}\times 100\%$$ (3)

## 3 Results

### 3.1 Identification results of the physical and chemical properties of artificial antigens

#### 3.1.1 UV identification

**Figure 4:**
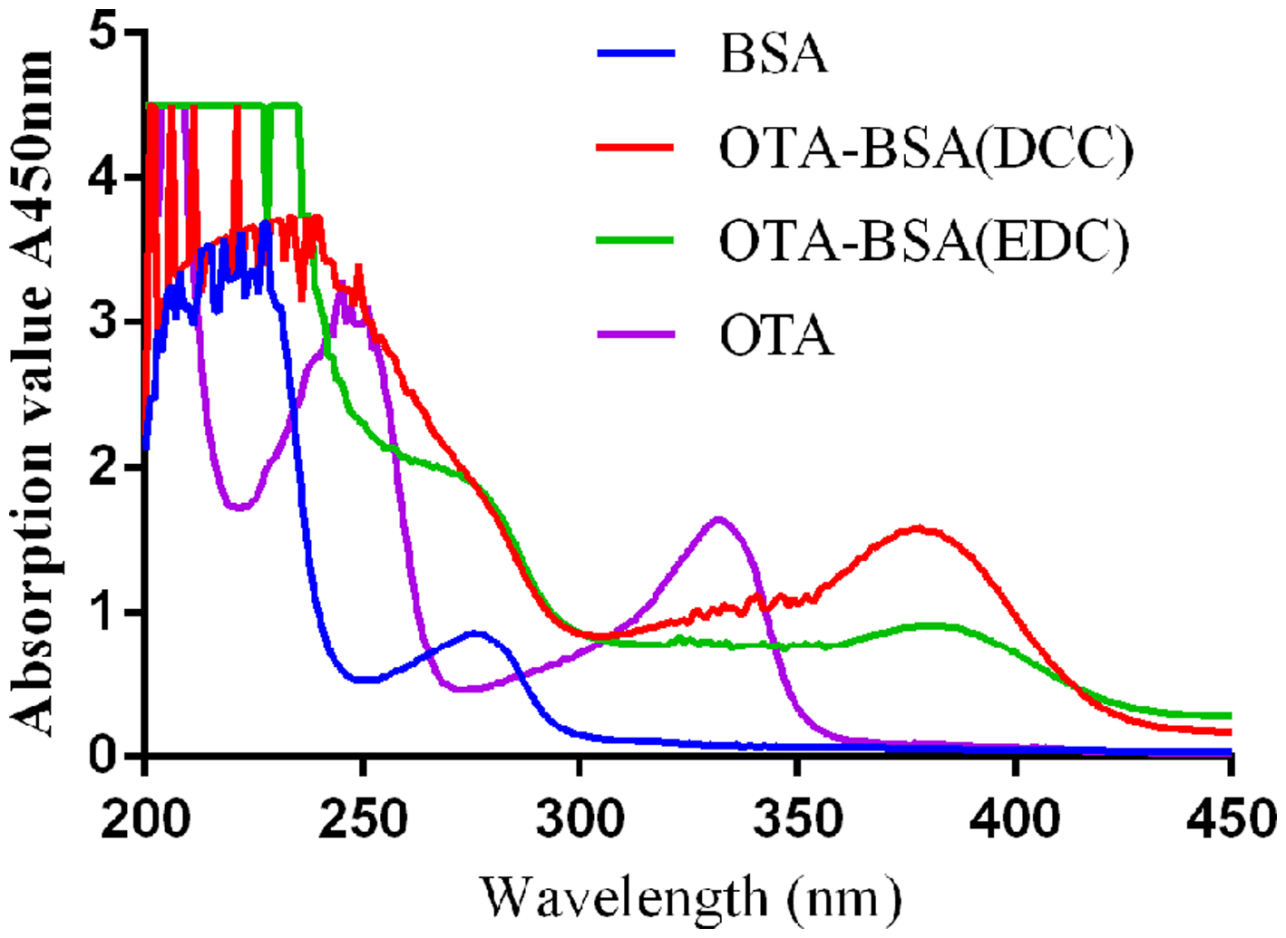
The UV identification of two artificial antigens.

UV scanning was performed on BSA, OTA, OTA-BSA (DCC), and OTA-BSA (EDC), and the results are shown in Figure 4. BSA exhibited a maximum absorption peak at 276 nm, whereas OTA exhibited a maximum ultraviolet absorption peak at 332 nm. The two artificial antigens (OTA-BSA [DCC] and OTA-BSA [EDC]) both demonstrated maximum absorption peaks at 378 nm, thereby indicating a shift relative to those of BSA and OTA. These findings prove that the artificial antigens that were prepared via both methods were successfully conjugated. UV scanning was used to obtain the absorbance values of BSA, OTA, and the conjugates (OTA-BSA [DCC] and OTA-BSA [EDC]), which were observed at 276 nm and 332 nm, respectively. The molecular binding ratios of OTA-BSA (DCC) and OTA-BSA (EDC) were calculated to be 10.88:1 and 7.84, respectively.

#### 3.1.2 IR identification

**Figure 5:**
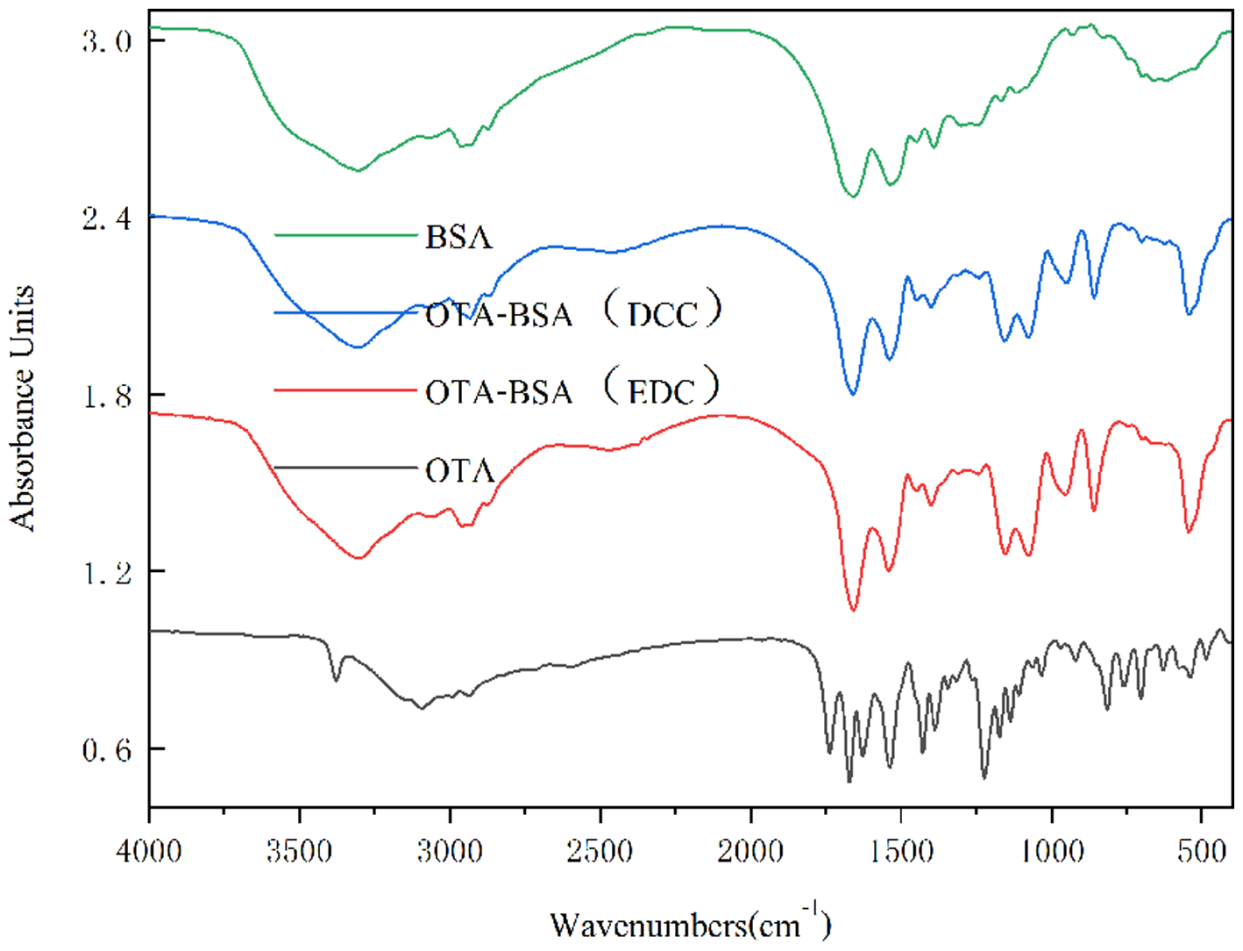
The IR identification of two artificial antigens.

The results are shown in Figure 5. Compared with BSA, the two artificial antigens (OTA-BSA [DCC] and OTA-BSA [EDC]) both exhibited amino acid characteristic peaks of the carrier protein BSA. Moreover, the IR spectra of these antigens exhibited similar absorption in the regions of 3,200 ~ 2,500 cm^−1^ and 1,660 ~ 1,500 cm^−1^, thus indicating the presence of BSA in both artificial antigens. Compared with OTA, the artificial antigens known as OTA-BSA (DCC) and OTA-BSA (EDC) exhibited similar absorption in the region of 1,150 ~ 1,050 cm^−1^, which is a characteristic peak of hydroxyl generation in OTA, whereas BSA demonstrates no absorption in this region, thereby indicating the presence of OTA in the artificial antigens. In addition, the hydroxyl group in OTA also produced sharp characteristic peaks in the range of 3,400 ~ 3,200 cm^−1^; however, the absorption peaks of the two artificial antigens in this area were masked by the amino acid characteristic peaks produced by BSA. The experimental results revealed that both artificial antigens were successfully conjugated.

### 3.2 Immunological characteristics of the artificial antigens

#### 3.2.1 Potency determination

**Figure 6:**
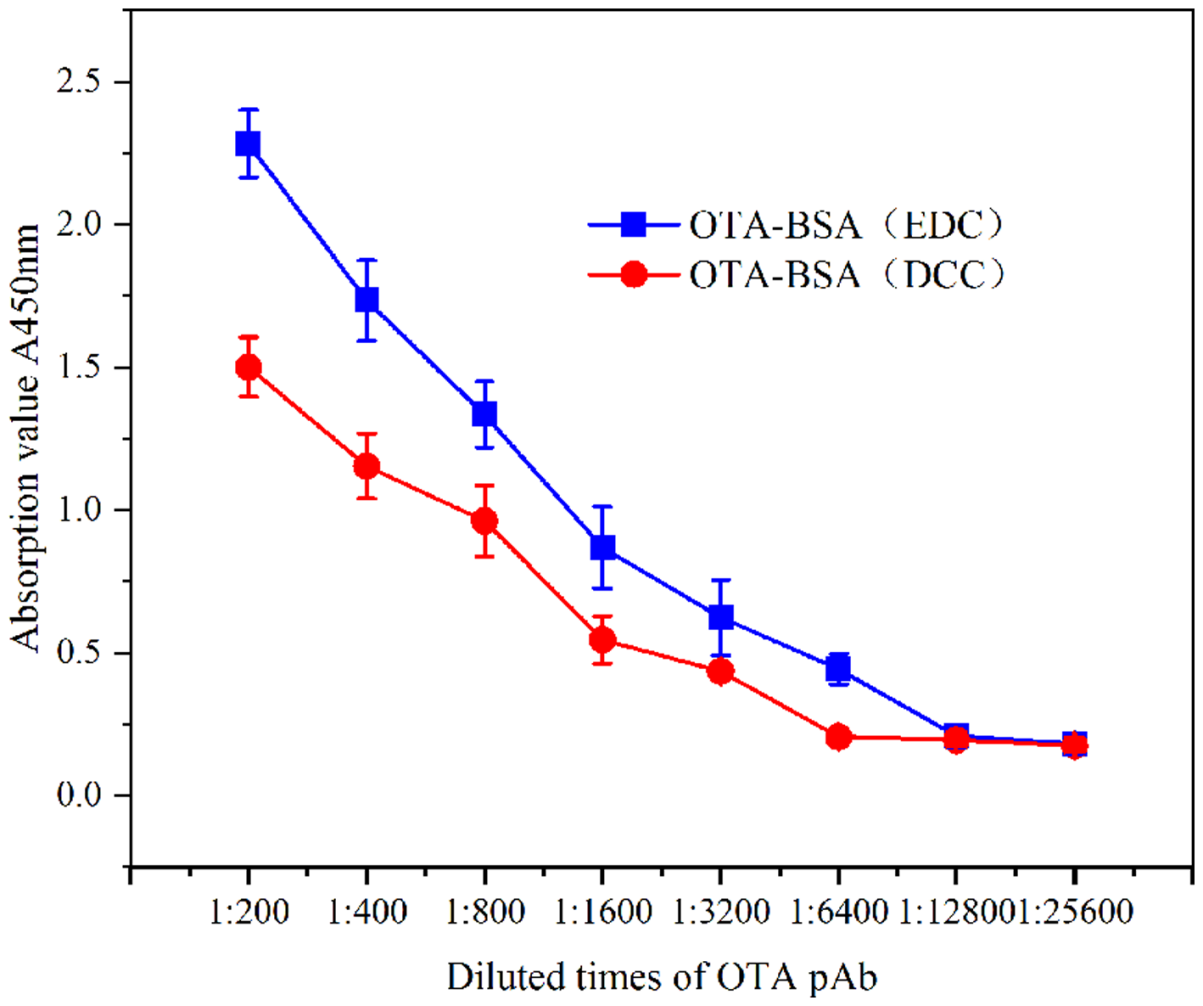
The titer curves of DON pAb identification.

The potency results are shown in Table 1 and Figure 6. Two groups of 6-week-old female BALB/c mice were immunized with two artificial antigens, with five mice being included per group. After 5 immunizations, the potency was measured via indirect ELISA. A single mouse exhibiting the highest potency was selected from each group, and a total of two mice were selected for comparison. All of the OTA pAb titers achieved a potency of 1:3.2 × 10^3^. The results demonstrated that the potencies of both artificial antigens met the potency requirements; however, when evaluated on the basis of immune efficacy, OTA-BSA (EDC) was superior to OTA-BSA (DCC).

**Table 1:** The titers determination of OTA pAb of two mice (*n* = 3).

Number	Diluted multiple of antiserum								Negative	Blank
	200	400	800	1,600	3,200	6,400	12,800	25,600		
OTA-BSA(EDC)	2.205	1.608	1.203	0.831	0.608	0.456	0.171	0.142	0.081	0.107
	2.418	1.886	1.420	1.027	0.765	0.489	0.247	0.212	0.118	0.126
	2.224	1.707	1.380	0.750	0.500	0.385	0.211	0.196	0.100	0.097
OTA-BSA(DCC)	1.400	1.215	1.106	0.633	0.436	0.186	0.171	0.188	0.090	0.115
	1.608	1.224	0.905	0.540	0.412	0.220	0.212	0.189	0.105	0.093
	1.494	1.024	0.876	0.467	0.460	0.214	0.204	0.150	0.103	0.106

#### 3.2.2 Sensitivity determination

**Figure 7:**
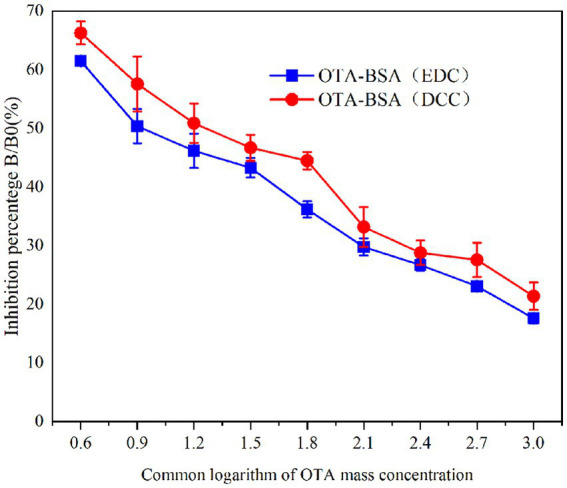
Inhibition curves of OTA pAb.

The sensitivity results are shown in Table 2 and Figure 7. The blocking ELISA curves of the two groups of mice demonstrated a good linear relationship, with the OTA-BSA (EDC) group exhibiting the best inhibitory effect. The linear regression equation was determined to be y = −17.222x + 68.2 (R^2^ = 0.982), and the IC_50_ was 11.40 ng/mL. The sensitivity of the OTA-BSA (DCC) group was observed to be slightly lower compared with the OTA-BSA (EDC) group.

**Table 2:** Regression equation, *R*^2^ and IC_50_ for OTA pAb.

Serial number	Regression equation	*R*^2^ value	IC_50_ (ng/ml)
OTA-BSA(EDC)	*y* = −17.222*x* + 68.2	0.982	11.40
OTA-BSA(DCC)	*y* = −18.183*x* + 74.619	0.979	22.59

#### 3.2.3 Specific identification

The identification results are shown in Table 3. The OTA pAb that was prepared from two artificial antigens was able to recognize OTA with 100% accuracy, among which the OTA-BSA (EDC) group exhibited the best sensitivity (with an IC_50_ of 11.40 ng/mL). With the exception of the high CR% values of 86.69 and 62.02% observed with OTB and OTC, respectively, the CR% values for the other compounds were less than 0.3%. The results indicated that the optimal artificial antigen for preparing antibodies with high sensitivity and specificity against OTA is the OTA-BSA (EDC) antigen.

**Table 3:** The Specific identification of pAb.

Compounds	OTA-BSA (EDC)		OTA-BSA (DCC)	
	IC_50_ (ng/mL)	CR (%)	IC_50_ (ng/mL)	CR (%)
OTA	11.40	100	22.59	100
OTB	13.15	86.69	28.79	78.46
OTC	18.38	62.02	34.32	65.82
DON	>8.0 × 10^3^	<0.3	>8.0 × 10^3^	<0.3
ZEN	>8.0 × 10^3^	<0.3	>8.0 × 10^3^	<0.3
AFB1	>8.0 × 10^3^	<0.3	>8.0 × 10^3^	<0.3

OTA: ochratoxin A; OTB: ochratoxin B; OTC: ochratoxin C; DON: deoxynivalenol; ZEN: zearalenone; AFB1: aflatoxin B1.

7 Inhibition curves of OTA pAb.

## 4 Discussion

### 4.1 Design of OTA hapten molecules

Current literature reports on OTA antigens have primarily focused on the use of the OTA molecule itself as the hapten. Artificial antigens are then obtained by coupling carrier proteins via the carboxyl group that is naturally present in the OTA molecule. For example, Zhang et al. (48) prepared artificial antigens and monoclonal antibodies for OTA by activating OTA molecules with DCC for protein coupling, and they achieved an IC_50_ of 7.6 ng/g for the antibody. Although these antibodies have been applied in the detection of actual clinical samples, complex matrices often require significant dilution to eliminate interference. Consequently, there is a need for more sensitive antibodies to establish more sensitive enzyme-linked immunoassay methods (49). Additionally, the use of the OTA molecule itself as a hapten has notable limitations. For example, the carboxyl carbon chain is too short, which hinders the complete exposure of the OTA molecule after coupling with carrier proteins. This partial obscuring of antigenic epitopes ultimately leads to a decrease in antibody affinity and sensitivity. By increasing the number of carboxyl carbon chains (typically, an increase of 3 to 6 carbons is considered reasonable), this issue can be effectively addressed. Therefore, we designed a structurally sound novel hapten. The OTA molecule contains a carboxyl group, thereby allowing for coupling via the mixed anhydride method or the active ester method. The mixed anhydride method often involves complex synthesis processes, with the production of numerous byproducts that are difficult to remove, thereby resulting in poor specificity of the obtained antibody sera. Conversely, the active ester method involves reacting the hapten with NHS to significantly improve the activation efficiency of the carboxyl group. Carrier proteins are subsequently added under the action of a coupling agent, which greatly reduces the self-crosslinking effect of protein molecules and enhances the coupling efficiency. Furthermore, the active ester method produces fewer byproducts and yields antibodies with strong specificity. Hence, this study utilized the active ester method for the preparation of the haptens (50). This prepared hapten is expected to facilitate the production of OTA antibodies exhibiting increased affinity and sensitivity, thereby providing a crucial molecular reagent for OTA immunoassay methods.

### 4.2 Preparation of OTA artificial antigens and molecular binding ratio analysis

In this study, a hapten was prepared via the active ester method. The carboxyl group in the OTA molecular structure was first activated by NHS to generate a reactive intermediate, which then underwent a condensation reaction with the amino group on the carrier protein under the action of the coupling agent DCC (or EDC) to produce an artificial antigen. Typically, for small-molecule haptens containing carbonyl or carboxyl groups, coupling methods such as CDI, EDC, IBCF, and NHS can be employed to conjugate the haptens with carrier proteins. These methods involve the formation of an amide bond (–CO–NH–) between the amino group (–NH_2_) in the carrier protein and the carbonyl (–C=O–) or carboxyl (–COO–) group in the hapten (51). In this study, two methods for preparing artificial antigens were compared, and the results indicated that the coupling effect under the action of EDC (as referenced and improved upon by Venkataramana, et al. (38), Guo et al. (39), Yexuan Ren et al. (40)) yielded the best results. DCC, which plays a profound role in peptide synthesis as a dehydrating agent, is a commonly used lipid-soluble condensing agent. However, it is prone to crosslinking when exposed to water and can only be dissolved in organic solvents. Additionally, NHS exhibits hygroscopicity. In this experiment, the coupling protein (BSA) needed to be dissolved in an aqueous solution, thus leading to difficulties in achieving anhydrous reaction conditions, which led to unsatisfactory results. Therefore, this experiment was suitable for the preparation of artificial antigens under the action of the coupling agent EDC. Furthermore, the simplicity and ease of operation of the EDC test scheme provide a low-cost solution for the rapid monitoring of OTA in feed.

However, there is significant debate regarding the optimal binding ratio. Based on a review of numerous studies in this field, we concluded that a binding ratio of 5 ~ 15:1 for OTA to BSA is more reasonable. Moreover, it is not true that an increased number of haptens coupled to the carrier corresponds to better results. According to previous reports, excessive hapten coverage on the carrier surface is not conducive to the binding of the carrier to lymphocyte antigen receptors, and it cannot stimulate the body to produce an effective immune response. Therefore, the determination of the molecular binding ratio before immunization is highly important. In this study, the binding ratios of OTA to BSA were 10.88:1 and 7.84:1, both of which are within the range of 5 ~ 15:1. However, 7.84:1 falls within the typical biomolecular binding ratio range (usually ≤10) and is close to the molecular binding ratio achieved in the method described by Yexuan Ren et al. (40). Immunized mice also achieved better titer, sensitivity, and specificity results. These results indicate that OTA could successfully bind to the carrier protein BSA. In terms of hapten molecular design and artificial antigen coupling, many empirical designs and predictive designs have been developed in the laboratory. Finally, the successful coupling of the artificial antigen was verified via physical and chemical identification, especially regarding the identification of immunological characteristics. Therefore, in artificial antigen coupling, it is necessary to utilize multiple preparation methods and compare the results by separately immunizing animals to select the best artificial antigen preparation method.

### 4.3 Analysis of the immunological characteristics of the OTA pAb

This study conducted an immunological analysis by determining the titer, sensitivity, and specificity of the OTA pAb. First, the titer of OTA pAb was measured via indirect ELISA. After five immunizations, one mouse exhibiting the highest titer was selected from each group for a total of two mice for comparison. The polyclonal antibody serum titer achieved a ratio of 1:3.2 × 10^3^. Metabolic poisoning caused by the immunization of mice with biotoxins is a long-term cumulative process; therefore, the polyclonal antibody serum titers of these mice are generally not too high. Therefore, the titers of both artificial antigens met the immunological requirements; however, OTA-BSA (EDC) was superior to OTA-BSA (DCC) in terms of the immunization effect. Second, the sensitivity of OTA pAb was determined via indirect competitive ELISA. The blocking ELISA curves of the two groups of mice demonstrated a good linear relationship, with the OTA-BSA (EDC) group of mice demonstrating the best inhibition effect. The linear regression equation was determined to be y = −17.222x + 68.2 (R^2^ = 0.982), and the IC_50_ was 11.40 ng/mL. Moreover, the OTA-BSA (DCC) group exhibited slightly lower sensitivity. Additionally, the specificity of the OTA pAb was identified via cross-reactivity tests. Both methods produced OTA pAbs that could recognize OTA with 100% accuracy. Among them, the OTA-BSA (EDC) method demonstrated the best sensitivity. In addition to the higher cross-reactivity rates of 86.69 and 62.02% observed with OTB and OTC, respectively, the cross-reactivity rates with the other compounds were less than 0.3%. Due to the high degree of similarity in molecular structure between OTB, OTC, and OTA, the cross-reactivity rates were relatively high. However, other toxins (such as DON, ZEN, and AFB1) exhibit significant structural differences compared to OTA, thus resulting in lower cross-reactivity rates. Therefore, this study systematically compared the effects of two strategies on OTA antigen performance for the first time, with an aim of preparing antibodies with high sensitivity and specificity toward OTA. The best artificial antigen was identified as OTA-BSA (EDC).

## 5 Conclusion

Based on the structural characteristics of the OTA molecule and referencing extensive literature, an artificial antigen preparation system utilizing two different coupling agents, DCC and EDC, was constructed via the N-hydroxysuccinimide active ester method. The properties of the two artificial antigens were systematically compared. Structural characterization was performed using techniques such as ultraviolet spectroscopy and infrared spectroscopy, and their immunogenicity was evaluated using a mouse immunization model. The results indicated that the artificial antigen prepared by the EDC method was superior to that prepared by the DCC method in terms of molecular conjugation ratio, immunogenicity, and detection sensitivity. The polyclonal antibody titer and specificity induced by the EDC-based antigen met the desired standards. Therefore, this study establishes a profound antigenic foundation for the subsequent development of highly sensitive OTA immunoassay products.

## Data Availability

The original contributions presented in the study are included in the article/supplementary material, further inquiries can be directed to the corresponding authors.

## Glossary

OTA: Ochratoxin A
OTB: Ochratoxin B
OTC: Ochratoxin C
DON: Deoxynivalenol
ZEN: Zearalenone
AFB1: Aflatoxin B1
NHS: N-hydroxysuccinimide active ester
BSA: Bovine serum albumin
OVA: Ovalbumin
DCC: N, N′-dicyclohexylcarbodiimide
EDC: 1-(3-dimethylaminopropyl)-3-ethylcarbodiimide hydrochloride
UV: Ultraviolet
IR: Infrared
pAbs: Polyclonal antisera
mAbs: Monoclonal antibodies
IC_50_: Half-maximal inhibitory concentration
THF: Anhydrous tetrahydrofuran
DMF: N, N-dimethylformamide
KBr: Potassium bromide
FIA: Freund’s incomplete adjuvant
FCA: Freund’s complete adjuvant
ELISA: Enzyme-linked immunosorbent assay
CR%: Cross-reactivity rate
MDPI: Multidisciplinary digital publishing institute
DOAJ: Directory of open access journals
TLA: Three letter acronym
LD: Linear dichroism
